# Androgens improve ovarian follicle function impaired by glucocorticoids through an androgen-IGF1-FSH synergistic effect

**DOI:** 10.3389/fendo.2022.951928

**Published:** 2022-10-19

**Authors:** Lingyun Gao, Hongna Gao, Wenjun Wang

**Affiliations:** ^1^ Department of Integrated Traditional & Western Medicine, Obstetrics and Gynecology Hospital of Fudan University, Shanghai, China; ^2^ Department of Integrated Traditional & Western Medicine, Shanghai Key Laboratory of Female Reproductive Endocrine Related Diseases, Shanghai, China

**Keywords:** glucocorticoids, androgen, IGF1, FSH, ovarian follicle

## Abstract

High concentrations of glucocorticoids caused by chronic stress are known to affect ovarian function and cause diminished ovarian reserve. Androgens are essential for early-stage ovarian follicle development, but the effects and mechanisms of androgens on follicle development under chronic stress remain unclear. In this study, we aim to investigate the effects of high concentrations of glucocorticoids on the function of *in vitro* cultured ovarian cells and mouse early-stage ovarian follicles and to validate the hypothesis that androgen–insulin-like growth factor 1 (IGF1)–follicle-stimulating hormone (FSH) synergistic signaling helps to ameliorate the damage caused by high concentrations of glucocorticoids. KGN cells (human granulosa cell line) and mouse primary cells were treated with different concentrations of glucocorticoids, and the cell proliferation, apoptosis, and sex hormone secretion were detected. The effects of glucocorticoid and androgens on IGF1 receptor (IGF1R) and FSH receptor (FSHR) expression in KGN cells were detected by Western blot. Steroidogenic synthase expressions under androgens and androgen-IGF1-FSH combination treatment were examined by qPCR after manipulation using low and high concentrations of glucocorticoids. The mechanism of androgen regulation of IGF1R and FSHR was explored by small interfering RNA (siRNA) and chromatin immunoprecipitation (ChIP)-qPCR. Damage of glucocorticoids and the treatment effects of androgens were further validated in mouse ovarian follicles cultured *in vitro*. The results demonstrated that prolonged treatment with high-dose glucocorticoids reduced cell viability of granulosa cells, inhibited their sex hormone secretion, and impaired their sensitivity to IGF1 and FSH signaling by affecting IGF1R and FSHR functions. Androgens at an appropriate dose range improved early-stage follicle development and their hormone secretion under high-dose glucocorticoid treatment, which was related to increased transcription of *Igf1r* and *Fshr*. This work showed that excessive glucocorticoids impaired ovarian function and validated that balanced concentrations of androgens synergized with IGF1 and FSH to improve the function of early-stage ovarian follicles under conditions of chronic stress.

## Introduction

Diminished ovarian reserve (DOR) is defined as a decrease in ovarian follicle number or oocyte quality and plays an important role in the etiology of female infertility (1). A recent study reports that DOR prevalence in infertile patients from *in vitro* fertilization centers in the United States increased from 19% to 26% from 2004 to 2011 (2). DOR is characterized by a decreased antral follicle count, elevation of serum follicle-stimulating hormone (FSH) concentration, and decreased serum estradiol (E2) and anti-Müllerian hormone (AMH) concentrations. Along with many other causes of DOR including genetic factors, environmental pollution, and infection, chronic stress is emerging in importance in modern society (3). Stress is commonly defined as a state of real or perceived threat to homeostasis that may challenge an organism’s wellbeing (4). The hypothalamic-pituitary-adrenal (HPA) axis, composed of the hypothalamus, pituitary gland, and adrenal glands, regulates the body’s response to stress (5). As the end product of the HPA axis (6), aberrant glucocorticoid release [i.e., cortisol (CORT) in humans, corticosterone (CORTN) in rodents (7)] can be damaging to the reproductive system owing to extreme or chronic stress exposure (8). However, studies reporting the adverse effect and underlying mechanism of elevated glucocorticoids on ovarian follicle development are still limited.

Insulin-like growth factor 1 (IGF1) and FSH signals are two important growth factors for folliculogenesis and increase with follicle maturation (9, 10). Studies in pigs have shown that direct exposure to high concentrations of glucocorticoids decreases ovarian IGF1 production both *in vitro (* 11) and *in vivo (* 12). In female mice, injection of glucocorticoids significantly decreases IGF1 transcription in granulosa cells and decreases E2 concentrations in both the serum and ovary (13). Our previous work revealed that protein expression of FSH receptors (FSHRs) is significantly decreased in a chronic unpredictable stress-induced DOR mouse model (14, 15). Such studies indicate that IGF1 and FSHR synthesis in ovaries can potentially be impaired by excess glucocorticoids and influence follicle maturation.

In recent decades, it has been recognized that a balanced concentration of androgens is essential for early-stage follicle development (16–18). Clinical studies have found that DOR patients possess a lower serum androgen concentration compared to those with a normal ovarian reserve (19). Our previous work found that serum androgen concentrations were also significantly reduced in DOR mice induced by chronic unpredictable stress (14). In addition, studies have found an inverse correlation between serum testosterone (T) and serum CORT concentrations in women (20). Decreased serum T levels affect the function of the hypothalamic neuroendocrine system and promote activation of the HPA axis, leading to abnormal stress responses and stress-related disorders (21). Lower levels of sex hormones are also related to lower negative feedback of glucocorticoids on the HPA axis (22). Therefore, a “vicious cycle” of high glucocorticoid and low androgen levels under conditions of chronic stress may exacerbate the impairment of early ovarian follicle development, leading to DOR.

Clinical studies suggest that supplementation with androgens or their precursor dehydroepiandrosterone (DHEA) in patients with low androgen levels can significantly preserve the ovarian reserve and improve egg retrieval rate and pregnancy outcome in DOR patients, but the specific mechanism remains to be elucidated (23, 24). In mouse ovarian follicles cultured *in vitro*, transcription of FSHR was significantly improved after T or dihydrotestosterone (DHT) treatment (25). The action of androgens is also suggested to be related to the expression of IGF1R in human granulosa cells from primordial and primary follicles (26). Moreover, it has been demonstrated in rodent studies that the actions of FSH on granulosa cell and follicle development depend on the presence of IGF1 and active IGF1R (27). These results suggest that androgen supplementation may ameliorate glucocorticoid-induced damage to ovarian follicle development through IGF1 and FSH signaling.

Collectively, the above findings suggest that excess glucocorticoids and low levels of androgens under chronic stress may potentially impair early-stage ovarian follicle development. Androgen supplementation may improve ovarian function under chronic stress by acting synergistically with IGF1 and FSH signaling. To test the hypothesis, the effects of glucocorticoids and androgens on a human granulosa-like tumor cell line (KGN cells), mouse granulosa cells (mGCs), mouse theca/interstitial cells (TICs), and mouse preantral follicles were observed. The underlying mechanism was verified by comparing the changes of IGF1R, FSHR, and steroidogenesis hormone synthase after androgen application and androgen-IGF1-FSH combined application.

## Materials and methods

### Experimental design

The study was composed of three phases. To first explore the effect of glucocorticoids on ovarian steroidogenesis cells, especially granulosa cells, KGN cells were cultured and treated with different concentrations of CORT for 24, 48, and 72 h. Cell viability, apoptosis, and hormone secretion of E2 and IGF1 were detected in different groups. To double the confirmation of the results, mGC and TIC were isolated and treated with CORTN over different time periods and E2 and T secretion were detected, respectively. To validate the mechanism of the reduced E2 secretion after high concentrations of glucocorticoid treatment, the expression of androgen receptor (AR), IGF1R, p-IGF1R, and FSHR was detected in KGN cells. The expression of IGF1R in mGC was observed after treatment with different concentrations of CORTN. Furthermore, according to a recent report, except for the canonical action through their nuclear receptor to regulate protein synthesis, glucocorticoids can exert a direct impact on some cell signals (28). Therefore, we also detected the phosphorylation activation levels of the steroidogenesis signaling molecules Protein kinase B (AKT) and extracellular signal-regulated kinase (ERK). In order to clarify the mechanism by which long-time exposure to high-dose glucocorticoid treatment inhibits the autophosphorylation of IGF1R in KGN cells, low-dose (0.1 μM) CORT and high-dose (1 μM) CORT were applied to KGN cells respectively for 48 and 72 h. The cells were collected for co-immunoprecipitation (co-IP) assay to detect the protein–protein interaction between glucocorticoid receptors (GRs) and IGF1R.

In the second phase, to validate our hypothesis of androgen-IGF1-FSH synergistic effect in ameliorating the impairment of high concentrations of glucocorticoids, we first verified the bidirectional regulation effect of androgens at 0–10-nM concentrations on the protein expression of IGF1R and FSHR in KGN cells after 12- and 24-h treatments. Based on the results, we adopted 10 nM as the “low dose” and 50 nM as the “high dose” and reconfirmed the bidirectional effect of androgens by treating KGN cells with FSH combined with low/high concentrations of androgens for 24 h. The messenger RNA (mRNA) expression difference of steroid hormone synthase in KGN cells was detected by qPCR. The synergistic effect of androgen-FSH-IGF1 signaling was verified by treating KGN cells with 0.1 µM CORT or 1 µM CORT for 24 h, followed by treatment with a combination of the three factors or treatment with one of the three factors for 48 h. The qPCR results of steroid hormone synthase expression were evaluated in the different groups. To further explore the mechanism of the synergistic effects of androgens with IGF1 and FSH, we transfected KGN cells with small interfering RNA (siRNA) to knock down the expression of ARs. The expression of ARs after transfection was validated by qPCR and Western blot (WB). Based on our further hypothesis that ARs may regulate the gene expression of *Igf1r* and *Fshr* as a transcription factor, the chromatin immunoprecipitation (ChIP)-qPCR technique was applied after treating KGN cells with androgens at 10 nM concentration for 1 h.

In the last phase of the study, we observed the growth rate and E2 secretion of *in vitro* cultured mouse preantral follicles with different factors added to their culture media, including low and high concentrations of CORTN, androgens, androgen-high concentration of CORTN combined treatment, and androgen–IGF1–high-concentration CORTN combined treatment. Through analysis of changes in follicle diameter and the enzyme-linked immunosorbent assay (ELISA) results of the hormone levels in different groups after 3 days of culture, we aimed to further verify our hypothesis in an *ex vivo* model.

### Drugs and reagents

The following drugs and reagents were used in the study: L-15 media, McCoy’s 5a, M199 media, α-MEM media, and DMEM/F12 media without phenol red (Gibco), fetal bovine serum (FBS; Sciencell), fetuin (MCE), insulin-transferrin-selenium solution (Sigma), type IV collagenase (Absin, abs47048003), DNase I (Sangon Biotech), bovine serum albumin (BSA; BioFroxx), T (Shanghai Standard Technology Co. Ltd.), DHT (Selleck, S4757), CORT (Selleck, S1696), CORTN (Selleck, S4752), RNA isolation kit, 4× reverse transcription Mix and Color SYBR Green qPCR Master Mix (EZBioscience, A0012), Lipofectamine 3000 and opti-MEM (Invitrogen), shAR vectors and negative control shRNA vectors (GeneChem), protein A/G magnetic beads (Bimake, B23201), normal rabbit IgG (CST, 2729), ELISA kits (E2, Labor Diagnostika Nord, Cat# FR E-2000, RRID : AB_2916329; T, Labor Diagnostika Nord, Cat# AR E-8000, RRID : AB_2916330; IGF1, R and D Systems Cat# DG100B, RRID : AB_2915951), cell counting kit-8 (CCK-8) reagent (DOJINDO), Annexin V fluorescein isothiocyanate/ propidium iodide (FITC/PI) apoptosis detection kit (BD), GR antibody (Cell Signaling Technology Cat# 12041, RRID : AB_2631286), IGF1R antibody (Cell Signaling Technology Cat# 3027, RRID : AB_2122378), p-IGF1R antibody (Cell Signaling Technology Cat# 3024, RRID : AB-331253), FSHR antibody (Bioss Cat# bs-20658R, RRID : AB_2916328), AKT antibody (Cell Signaling Technology Cat# 9272, RRID : AB_329827), p-AKT antibody (Cell Signaling Technology Cat# 4051, RRID : AB_331158), ERK1/2 antibody (Cell Signaling Technology Cat# 4695, RRID : AB_390779), p-ERK1/2 antibody (Cell Signaling Technology Cat# 4370, RRID : AB_2315112), AR antibody (Santa Cruz Biotechnology Cat# sc-7305, RRID : AB_626671), CYP11A1 antibody (Proteintech Cat# 13363-1-AP, RRID : AB_2088552), AMH antibody (Abcam Cat# ab103233, RRID : AB_10711946), anti-Rabbit IgG antibody (Cell Signaling Technology Cat# 4414, RRID : AB_10693544), and anti-Mouse IgG antibody (Jackson ImmunoResearch Labs Cat# 115-005-008, RRID : AB_2338449). All first antibodies were diluted at 1:1,000.

### Animals

Six-week-old C57BL/6 male and female mice were purchased from Shanghai SLAC Laboratory Animal Ltd. After adaptive feeding for 1 week in the specific pathogen-free (SPF) facility, they were used to breed next-generation mice. Female C57 mice, aged 14–16 days, were bred for preantral follicle isolation. Female C57 mice aged 21 days were used to extract mGC and TIC. All experimental procedures followed the Criteria of the Medical Laboratory. Animal studies were reviewed and approved by the Animal Experimental Ethical Committee of Fudan University.

### Isolation and culture of mouse granulosa cells and theca/interstitial cells

Twenty-one-day-old female C57 mice were sacrificed by cervical dislocation. The bilateral ovaries were collected and kept in ice-cold L-15 media supplemented with 100 U/ml penicillin-G and 100 µg/ml streptomycin (LSP). The ovaries were washed with LSP twice and were punctured using 25G needles in a 35-mm dish on ice. The ovaries were extensively punctured to ensure that granulosa cells were released from follicles. The tissue-cell mixture was filtered through 100-µm and 40-µm Falcon mesh to screen out large oocytes and tissue remnants from granulosa cells. The suspensions were centrifuged at 1,000 rpm for 5 min and resuspended in McCoy’s 5a culture media containing 5% FBS and seeded to 12-well plates. The remaining tissue was washed with LSP twice and resuspended with 0.2 ml M199 media containing 2 mg/ml type IV collagenase and 1 mg/ml DNase I per ovary. The mixture was digested at 37°C for 1 h, during which the tissue mixture was pipetted >20 times every 15 min. The digestion was aborted by adding 5% FBS McCoy’s 5a media, and the dispersed cells were centrifuged at 1,000 rpm for 5 min. Cell pellets were resuspended with TIC culture media and seeded on 12-well plates. All cells were cultured under conditions of 37°C and 5% CO_2_. Cells were starved for 12 h before drug treatment. The upper range of CORTN concentration chosen in this study referred to the serum CORTN concentration results observed in mice under chronic unpredictable stress in previous studies (7, 14).

### KGN cell culture and treatment

KGN cells (human granulosa-like tumor cell line) were purchased from Guangzhou Saiku Biotechnology Co., Ltd., and identified using the short tandem repeat (STR) technique. KGN cells were cultured in Dulbecco's Modified Eagle Medium/Ham's F-12 Nutrient Mixture (DMEM/F12) media without phenol red containing 10% FBS. Culture medium was changed every 2 days. For drug treatments, cells were digested and seeded on six-well plates. After cell adhesion and achievement of a suitable density, the culture medium was replaced with DMEM/F12 and starved for 12 h before drug treatment. The upper range of CORT concentration chosen in this study referred to the serum CORT concentration results observed in previous clinical studies (29, 30). Androgen concentrations were chosen with the upper range above the diagnosis criteria of polycystic ovarian syndrome (31) and with the lower range below the serum androgen concentrations of DOR patients (19).

### Preantral follicle isolation and three-dimensional culture

The method of mouse preantral follicle isolation and three-dimensional (3D) culture was modified based on previous reports (32–35). Female mice aged 14–16 days were sacrificed by cervical dislocation and placed in 75% alcohol for sterilization for 10 min. The bilateral ovaries were collected in a sterile manner and placed in L-15 media containing 1 mg/ml BSA. The redundant fallopian tubes and fat around the ovaries were carefully removed under a stereomicroscope. The ovaries were transferred to a new 35-mm dish containing 1 ml α-MEM media supplemented with 2 mg/ml collagenase and 1 mg/ml DNase. Dishes were placed at 37°C for 10 min. After being washed with L-15 media twice, the ovaries were placed in a new 35-mm dish containing 1 ml L-15 media supplemented with 1 mg/ml BSA. Two insulin needles were used to carefully dissect secondary follicles from the ovaries under a stereomicroscope. The follicles were selected according to the following criteria: 100–130 µm diameter; intact basal layer with some theca cells attached; and a clear oocyte in the middle of the follicle. Selected follicles were transferred to α-MEM media containing 1% BSA for 1 h at 37°C.

**Figure 1:**
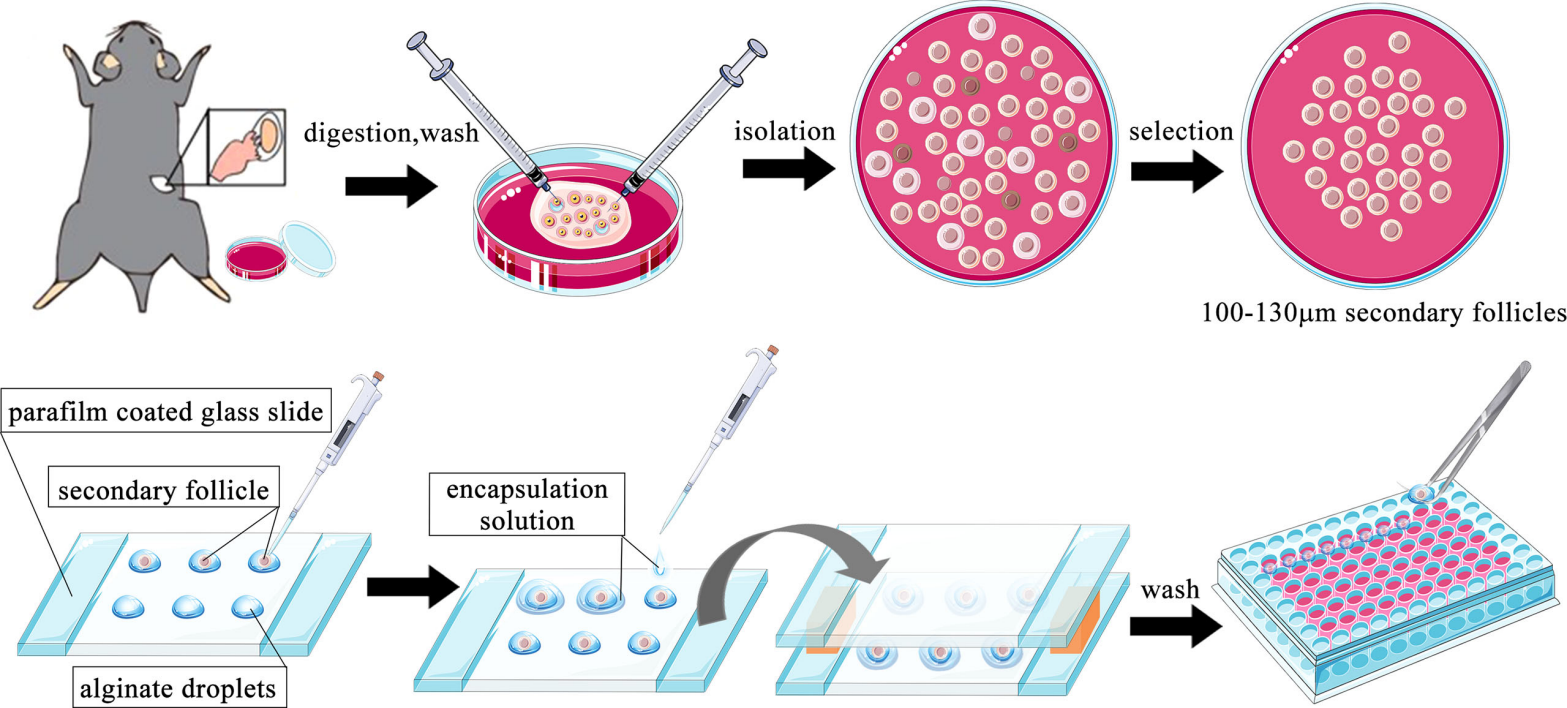
Schematic diagram of the follicle isolation and three-dimensional (3D) culture steps.

Alginate solution [1% (m/v)] was prewarmed to 37°C, and 7.5 µl drops were pipetted onto a glass slide wrapped with parafilm and a 3-mm spacer on each side. Each follicle was transferred into an alginate drop with the minimum volume of media. A solution containing 40 mM CaCl_2_ 150 mM NaCl was dropped onto the alginate drops, quickly followed by another parafilm-wrapped glass slide to cover the drops. The two slices were placed upside down, and left for 2 min to allow the gel to solidify. The follicle-containing alginate beads were washed once in follicle culture media (α-MEM supplied with 3 mg/ml BSA, 1 mg/ml fetuin, 5 µg/ml insulin-transferrin-selenium solution, 100 U/ml penicillin-G, 100 µg/ml streptomycin, and 100 ng/ml FSH). Each bead was transferred into one well of a 96-well plate containing 100 µl of culture media. The follicles were cultured in an incubator at 37°C and 5% CO_2_. The cultured follicles were examined daily under a light microscope, and diameters were measured using Image-Pro Plus software. After a 3-day culture, the culture medium was collected and an ELISA kit was used to detect E2 concentrations. All follicle culture experiments were repeated three times with at least 10 follicles contained in each group. Supernatants from 3–4 follicles were pooled together as one ELISA sample in each group. A brief flowchart of the process is shown in **Figure 1**. The concentration of DHT chosen for this study was based on a previous study in which the effect of a wide dose range of different types of androgens was observed on mouse follicles cultured *in vitro (* 36).

### Cell transfection

Lipofectamine 3000 was used. AR plasmid vector (2.5 µg) or control vector was mixed with 125 µl Opti-MEM medium. Lipofectamine 3000 reagent (7.5 µl) was diluted with 125 µl Opti-MEM medium. The two mixtures were combined and incubated for 10–15 min at room temperature (RT). Each mixture was used to transfect KGN cells for 6 h followed by normal culture media treatment for 48 h. Subsequently, cells were collected for RNA or protein analysis.

### Determination of estradiol, testosterone, and insulin-like growth factor 1 concentrations

Cell culture supernatant was collected after a 72-h intervention and was centrifuged at 4°C and 3,000 g for 15 min. The cell supernatant was detected for E2, T, and IGF1 using ELISA kits following the manufacturer’s instructions. Briefly, the culture supernatant was centrifuged to remove all unsolvable pellets. Serially diluted standard reagents and diluted samples were prepared as instructed. Standard reagents and samples were added to 96-well plates, followed by enzyme conjugate being added to each well. The plate was incubated in a specific temperature environment and washed several times with wash buffer. After addition of the substrate solution, the plate was incubated in the dark and the reaction was stopped with stop solution. The absorbance of each well was read at 450 nm by a plate reader (Biotek Multisken MK3). The concentrations were calculated using a standard curve. The analytic sensitivity for E2 was 10.6 pg/ml with an intra-assay and inter-assay of variation of <9.2% and 14.9%, respectively. The lowest analytical detectable concentration of T was 0.066 ng/ml with an intra-assay and inter-assay variability of <11.3%. The minimum detectable concentration of IGF1 ranged from 0.004 to 0.022 ng/ml with an intra-assay and inter-assay precision of <6.2%.

### Cellular RNA extraction and real-time PCR

Cells were washed in twice. RNA was extracted using an RNA isolation kit. The RNA concentration was determined by NanoDrop (Thermo Scientific). RNA (1 µg) was mixed with reverse transcription mix and diethyl pyrocarbonate (DEPC) water and reverse transcribed into cDNA under the following conditions: one cycle at 42°C for 15 min and one cycle at 95°C for 30 s. cDNA was diluted and mixed with primers, diethyl pyrocarbonate (DEPC) water, and SYBR Green Mix. RT-PCR reactions were performed under the following conditions: one cycle at 50°C for 2 min, one cycle at 95°C for 5 min, followed by 40 cycles at 95°C for 10 s and 60°C for 30 s. Data were normalized by the B2m level and calculated using the 2^-ΔΔCt^ method. The primer sequences are listed in **Table 1**.

**Table 1:** Primer sequences of target genes.

Species	Gene	Direction	Sequence
Human	*B2m*	Forward	GCTGGCGGGCATTCCTGAAG
		Reverse	AGAGCGGGAGGGTAGGAGAGAC
Human	*Igf1r*	Forward	GTTGGTGATTATGCTGTACGTC
		Reverse	TCCTTCATAGACCATCCCAAAC
Human	*Fshr*	Forward	GCATTCAATGGAACCCAACTAG
		Reverse	CGTGGAAAACATCATTAGGCAA
Human	*Star*	Forward	CATGGAGAGGCTCTATGAAGAG
		Reverse	GGACCTTGATCTCCTTGACATT
Human	*Cyp11a1*	Forward	TTTGAGTCCATCACTAACGTCA
		Reverse	GGTAGATGGCATCAATGAATCG
Human	*Cyp19a1*	Forward	GACTTTGCCACTGAGTTGATTT
		Reverse	CGATCAGCATTTCCAATATGCA
Mouse	*B2m*	Forward	TTCTGGTGCTTGTCTCACTGA
		Reverse	CAGTATGTTCGGCTTCCCATTC
Mouse	*Cyp11a1*	Forward	AGGTCCTTCAATGAGATCCCTT
		Reverse	TCCCTGTAAATGGGGCCATAC
Mouse	*Cyp19a1*	Forward	ATGTTCTTGGAAATGCTGAACCC
		Reverse	AGGACCTGGTATTGAAGACGAG
Mouse	*Lhr*	Forward	AATGAGTCCATCACGCTGAAAC
		Reverse	CCTGCAATTTGGTGGAAGAGA
Mouse	*Fshr*	Forward	CCTTGCTCCTGGTCTCCTTG
		Reverse	CTCGGTCACCTTGCTATCTTG

B2m, beta 2 microglobin; Igf1r, insulin-like growth factor 1 receptor; Fshr, follicle-stimulating hormone receptor; Star, steroidogenic acute regulatory protein; Cyp11a1, cytochrome P450 family 11 subfamily A member 1; Cyp19a1, cytochrome P450 family 19 subfamily A member 1; Lhr, luteinizing hormone receptor.

### Annexin V fluorescein isothiocyanate/ propidium iodide (FITC/PI) flow cytometry

Cell apoptosis was analyzed by flow cytometry using the Annexin-V/PI staining method. After treatment with different doses of glucocorticoids, KGN cells were harvested, washed twice with cold PBS, resuspended in 100 µl binding buffer, and stained with 5 µl Annexin V-FITC and 5 µl PI. The cells were incubated for 15 min in the dark and at RT before being analyzed by a flow cytometer (Beckman Coulter).

### Western blot analysis

Cells in six-well plates were washed in PBS twice and were lysed on ice with 80 µl radioimmunoprecipitation assay (RIPA) buffer containing PMSF and protease inhibitor cocktail for 10 min. Cells were scraped into 1.5 ml Eppendorf tubes and kept on ice for 30 min, then centrifuged at 4°C and 12,000 rpm for 20 min. The supernatant was transferred to a new tube. The protein concentration was determined by a bicinchoninic acid (BCA) kit. The protein solution was combined with Sodium Dodecyl Sulfate PolyAcrylamide Gel Electrophoresis (SDS-PAGE) loading buffer and boiled for 5 min at 95°C. Polyacrylamide gel (10%) was used to run the SDS-PAGE. Equal amounts of 25 µg protein samples were loaded onto the gel and transferred to polyvinylidene fluoride membranes by electrophoresis. The membrane was blocked in skim milk at RT for 1 h and then incubated in first antibody solutions at 4°C overnight. After being washed three times with tris buffered saline with Tween-20 (TBST) for 10 min for each replicate, the membranes were incubated in second antibody solutions at RT for 1 h. Excess antibodies were washed off three times with TBST for 10 min for each replicate. An enhanced chemiluminescent substrate kit was used to detect the immunoreactive bands using an ImageQuant LAS 4000 mini system. Relative quantitative protein expression was determined using ImageJ, version 1.51k (NIH, USA), by normalizing to the glyceraldehyde-3-phosphate dehydrogenase (GAPDH) internal control.

### Co-immunoprecipitation

KGN cells were lysed with RIPA buffer added with PMSF and protease inhibitor cocktail on ice for 20 min. GR antibody (1 µg) was added to the lysate sample and incubated on a rotating platform overnight at 4°C. Protein A/G magnetic beads (30 µl) were added to 1 ml of antibody–antigen lysate. The mixture was incubated on a rotating platform for 1 h at 4°C. The tubes were placed on a magnetic separation rack and rested for 1 min for the solution to be clear. The supernatant was discarded, and the beads were washed with 500 µl lysis buffer and placed on a magnetic rack for separation again. The wash step was repeated five times. The samples were kept on ice throughout the procedure. The final wash aimed to remove all trace substances from the beads. The beads were resuspended in 1× SDS-PAGE loading buffer and placed in an iron bath for 5 min at 95°C with a 300-rpm rotation. The denatured protein complex was released from the beads and was separated by a magnetic rack. The supernatant was collected for further WB analysis.

### Chromatin immunoprecipitation

KGN cells were cultured in a 14-cm dish (80% confluent) in 20 ml of DMEM + 10% FBS. DHT (10 nM) was added to the culture media for 1 h before the ChIP experiment. Formaldehyde (540 µl of 37%) was added to the media and incubated for 10 min at RT. Cross-linking was stopped by adding 2 ml of 10× glycine followed by incubation for 5 min at RT. The cells were washed with ice-cold PBS + protease inhibitor cocktail twice and scraped into a 1.5-ml tube. After centrifuging at 1,000 g for 5 min at 4°C, the cell pellet was resuspended with cell lysis buffer containing the protease inhibitor cocktail. After a 15-min incubation on ice, the tube was centrifuged again and the pellet was resuspended with nuclear cell lysis buffer containing the protease inhibitor cocktail. The cell lysate was then sonicated under high power, 30 s On 30 s Off, 10 cycles for three repeats. Sheared chromatin was centrifuged at 10,000 g at 4°C for 10 min, and the clear supernatant was collected for IP. Two aliquots of 50 µl of chromatin solution, namely, “AR” and “IgG,” were supplemented with 450 µl dilution buffer containing a protease inhibitor cocktail. Aliquots (5 µl) of the supernatant were saved at 4°C as “input” for further use. AR antibody (5 µg) was added to the “AR” sample, and 1 µg of mouse normal IgG was added to the “IgG” sample. Both tubes were also supplemented with 20 µl protein A/G magnetic beads and incubated overnight at 4°C with rotation. The beads were pelleted with a magnetic separator and washed with low-salt wash buffer, high-salt wash buffer, lithium chloride wash buffer, and Tris-EDTA buffer once, serially. The protein/DNA complexes were released from the beads by adding ChIP elution buffer and incubated at 62°C for 2 h with shaking, followed by being incubated at 95°C for 10 min. Immunoprecipitated chromatin was separated from the beads in a magnetic separation rack, and DNA was purified using spin columns. Immunoprecipitation of AR-associated DNA fragments was verified by qPCR using primers directed against *Igf1r* and *Fshr*. The primer sequences used for ChIP-qPCR are listed in **Table 2**. The values from the immunoprecipitated samples were normalized to that from the input DNA.

**Table 2:** Primer sequences for ChIP-qPCR.

Species	Gene	Direction	Sequence
Human	*Igf1r*	Forward	GGAACATCCAAAACTGAAACTCTT
		Reverse	TTCATATGATGGGATGGTTTTG
Human	*Fshr*	Forward	GAAGGAGGATCCAGGGAAAG
		Reverse	ACAGGAGGGCAGAGGAAAAT

### Statistical analysis

Concentrations are expressed as mean ± SEM. Statistical analysis was carried out with SPSS 22.0 software. t-tests were used to compare differences between two groups with equal variance. One-way ANOVA was used to compare differences between more than two groups. Two-way ANOVA was used to compare the effect of different concentrations of CORTN and treatment time on the secretion of T in TIC culture supernatant. The comparison of ovarian follicle diameter changes over 3 days between groups was made using repeated-measures ANOVA. P < 0.05 was taken to indicate a significant difference.

## Results

### Cortisol affected the viability and estradiol secretion of KGN cells in a time- and dose-dependent manner

**Figure 2:**
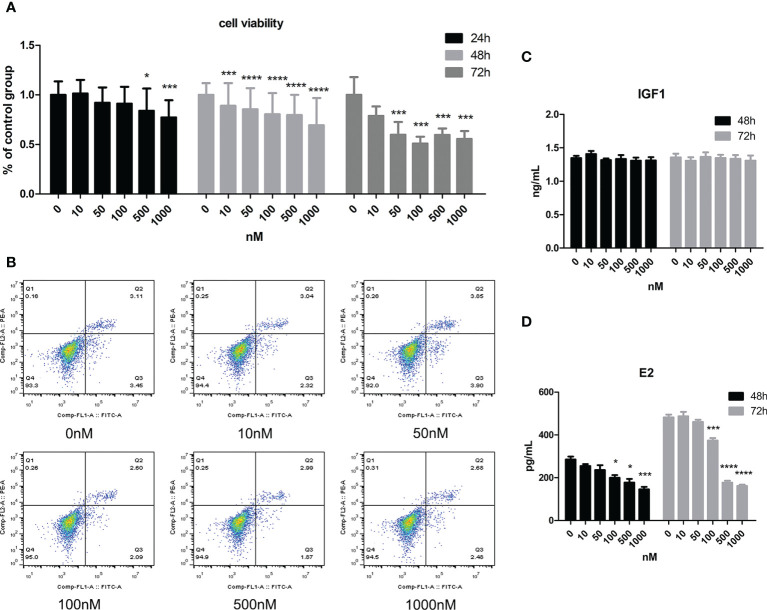
The impact of CORT at different doses on cell viability, apoptosis, E2 secretion, and IGF1 secretion. KGN cells were treated with solvent or 10, 50, 100, 500, and 1,000 nM CORT, respectively. Cell viability was assessed at 24, 48, and 72 h by the CCK-8 method. n ≥ 17 in each group at each time point; one-way ANOVA. **(A)** Cell apoptosis was determined at 72 h by flow cytometry. n = 3 with two repeats in each assay; one-way ANOVA. **(B)** IGF1 **(C)** and E2 **(D)** concentrations were determined by ELISA after treatment for 48 and 72 h n = 3 with one repeat in each assay; one-way ANOVA. *P < 0.05, ***P < 0.001, ****P < 0.0001 compared to group “0.”

As **Figure 2A** shows, cell viability decreased with increasing CORT dose. When the dose of CORT reached >500 nM and KGN cells were treated for 24 h, cell viability decreased significantly compared to that of the control group. Cell viability decreased more obviously when the treatment time was increased to 48 and 72 h. KGN cells were treated with CORT at different doses for 72 h followed by examination of apoptosis. As shown in **Figure 2B**, there was no significant difference in cell apoptosis among the groups. The cell culture supernatant of KGN cells after treatment with CORT at different doses after 48 and 72 h was collected and detected for E2 and IGF1 concentrations. **Figure 2C** shows no significant difference in IGF1 concentrations between different groups, but the E2 concentrations significantly decreased in the high-dose CORT groups (≥100 nM) compared to the solvent groups after 48 and 72 h (**Figure 2D**). The degree of decrease in E2 concentration increased with treatment time.

### Long-time exposure to high doses of corticosterone decreased estradiol secretion of mouse granulosa cells and testosterone secretion of theca/interstitial cells

**Figure 3:**
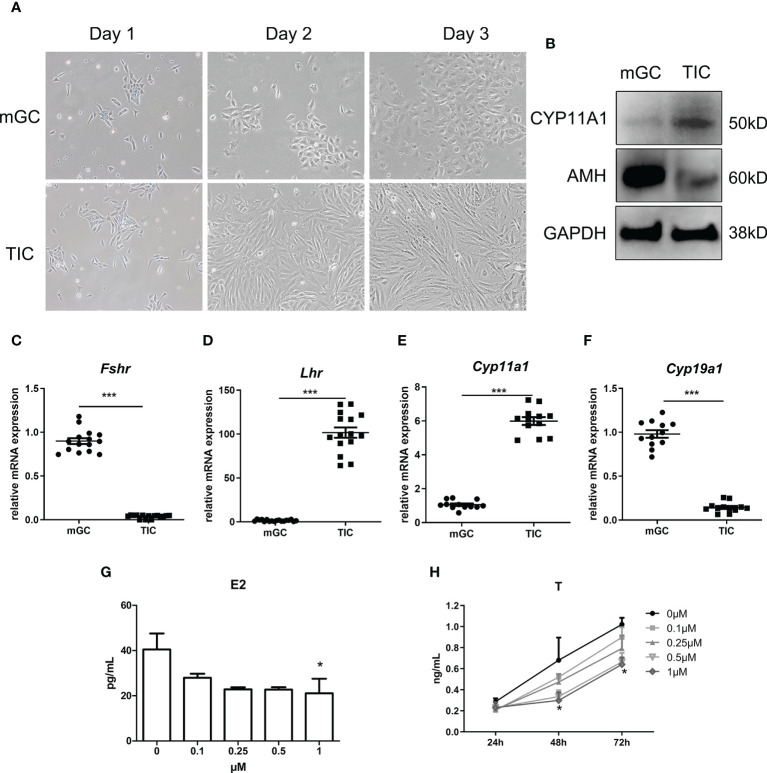
Identification of mGC and TIC and the influence of CORTN on sex hormone secretion of the two cell types. Morphology of the primary cell was recorded for 3 days after isolation using an inverted microscope. **(A)** Protein and RNA were extracted from the cells. CYP11A1 and AMH protein expression of the two cell types were examined by Western blot. n = 3; t-test. **(B)** Relative mRNA expression of *Fshr*, *Lhr*, *Cyp11a1*, and *Cyp19a1* was examined by qPCR. n = 15; t-test. **(C–F)** mGC and TIC were treated with solvent or 0.1, 0.25, 0.5, and 1 µM CORTN, respectively. Cell culture supernatant of mGC after 72 h was collected to determine the concentration of E2 by ELISA. n = 3 with one repeat in each assay; one-way ANOVA. **(G)** Cell culture supernatant of T after 24, 48, and 72 h was collected to determine the concentration of T by ELISA. (H) *P < 0.05, ***P < 0.001 compared to group “0.” n = 3 in each group at each time point with one repeat in each assay; two-way ANOVA.

We first identified the two cell types by morphology and the expression of specific markers using qPCR and WB. As shown in **Figure 3A**, mGC exhibited a polygonal and paving stone-like shape under the microscope, while TIC showed a spindle shape and cord-like shape; these findings were consistent with the morphological manifestations of epithelial cells and mesenchymal cells. **Figure 3B** shows that the protein expression of AMH and Cytochrome P450 Family 11 Subfamily A Member 1 (CYP11A1) in mGC was significantly higher than those in TIC. Furthermore, **Figures 3C–F** shows that the mRNA expression of *Fshr* and *Cytochrome P450 Family 19 Subfamily A Member 1 (Cyp19a1)* in GCs was significantly higher than that in TIC. *Cyp11a1* and *luteinizing hormone receptor (Lhr)* mRNA expression was significantly higher in TIC than that in mGC, which agrees with the expression of markers of the two cell types. CORTN at different doses was applied to mGC and TIC for 72 h. As shown in **Figure 3G**, E2 secretion decreased with the increase of CORTN concentration. The secretion of E2 after the 1-μM CORT treatment for 72 h was significantly lower than that of the control group. T secretion of TIC also decreased with the increase of CORTN concentration (**Figure 3H**). T level in the 1-µM CORTN group was significantly lower than that in the control group after 48 and 72 h of treatment.

### Long-time exposure to high-dose glucocorticoids decreased the responsiveness of granulosa cells to follicle-stimulating hormone and insulin-like growth factor 1 signals by decreasing their receptor number

**Figure 4:**
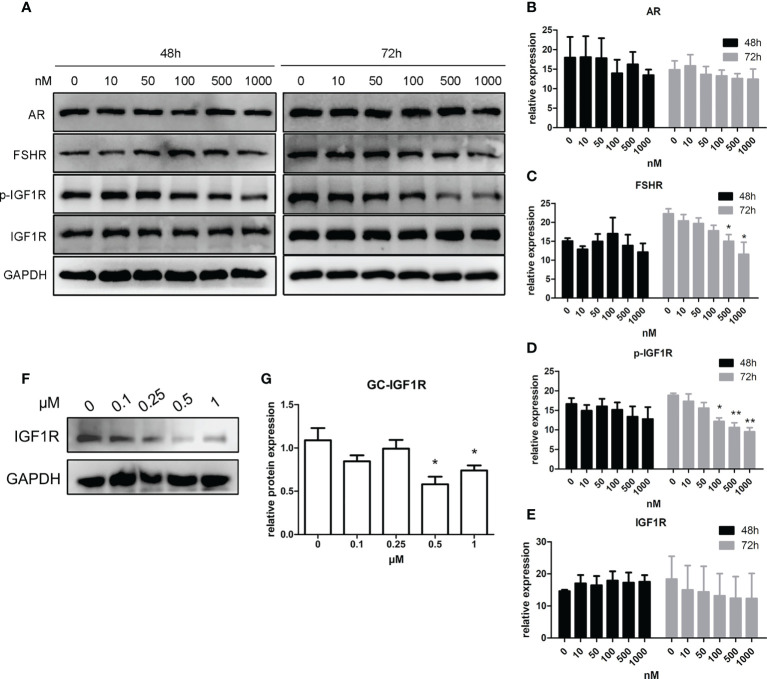
Impact of glucocorticoids at different doses on the protein expression of hormone receptors of KGN cells and mGC. KGN cells were treated with solvent or 10, 50, 100, 500, and 1,000 nM CORT, respectively. Protein levels of AR, FSHR, IGF1R, and p-IGF1R were determined by Western blot after 48 and 72 h **(A)** Semiquantitative analysis was performed after three repetitions. n = 3 in each time point and each group; one-way ANOVA. **(B–E)** mGCs were treated with solvent or 0.1, 0.25, 0.5, and 1 µM CORTN, respectively, for 72 h Cells were harvested for Western blot analysis of IGF1R **(F)** and semiquantitative analysis. n = 3; one-way ANOVA. **(G)** *P < 0.05, **P < 0.01 compared to group “0.”

As shown in **Figures 4A–E**, after a 48-h manipulation of CORT, the protein expressions of FSHR, p-IGF1R, and IGF1R were not significantly changed in KGN cells. However, when the treatment time was extended to 72 h, FSHR expression significantly decreased in the 500-nM CORT group and 1,000-nM CORT group compared to that of the control group. The protein expression of IGF1R remained unchanged, but p-IGF1R protein expression decreased significantly in 100-nM–1,000-nM CORT groups compared to that of the control group. mGC was cultured and treated with CORTN at different doses for 72 h. As shown in **Figures 4F, G**, IGF1R protein expression was significantly decreased in 0.5-µM and 1-µM CORTN groups compared to that of the control group.

### Long-time exposure to high-dose glucocorticoids hindered the interaction between glucocorticoid receptor and insulin-like growth factor 1 receptor in KGN cells

**Figure 5:**
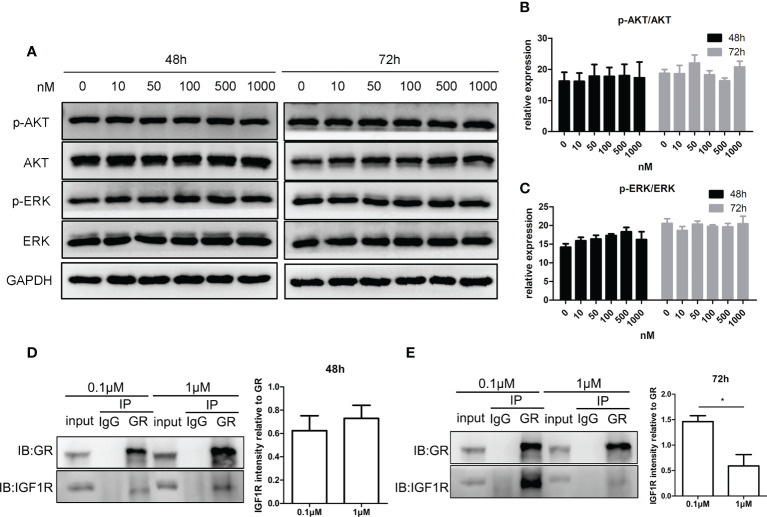
Impact of glucocorticoids on cell signals of steroidogenesis and the protein interaction between GR and IGF1R in KGN cells. KGN cells were treated by different doses of CORT for 48 and 72 h, respectively, and harvested for Western blot analysis of AKT, p-AKT, ERK, and p-ERK. **(A)** Phosphorylation levels of AKT **(B)** and ERK **(C)** were determined to assess the direct influence of glucocorticoids on the steroidogenesis signal. n = 3 in each time point and each group; one-way ANOVA. KGN cells were treated with low-dose (0.1 µM) and high-dose (1 µM) CORT, respectively. Protein–protein interaction between GR and IGF1R was detected by Co-IP after 48 h **(D)** and 72 h **(E)**. n = 3, t-test. *P < 0.05.

**Figures 5A–C** show that the phosphorylation level of AKT and ERK after treatment with CORT at various concentrations was almost the same. **Figures 5D, E** show that at 48 h, there was a positive interaction between GR and IGF1R, and there was no significant difference between the two CORT dose groups. However, after 72 h of treatment, the interaction between GR and IGF1R decreased significantly in the high-dose CORT group compared to that in the low-dose CORT group.

### An appropriate dose range of androgens increased the expression of insulin-like growth factor 1 receptor and follicle-stimulating hormone receptor in granulosa cells

**Figure 6:**
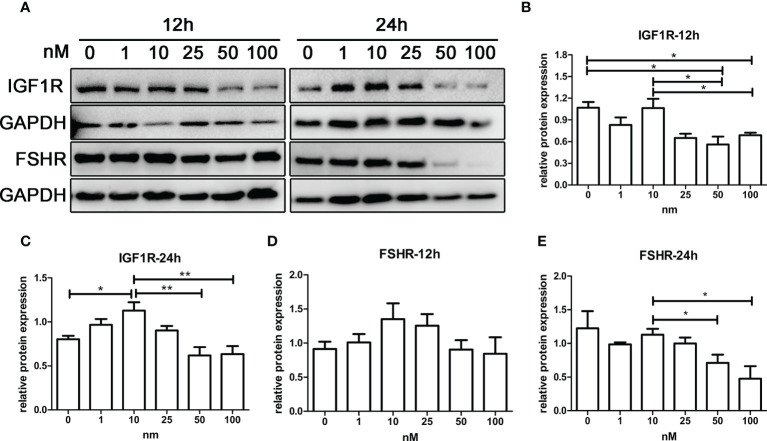
The effect of androgens on the expression of FSHR and IGF1R of KGN cells. KGN cells were treated with solvent or 1, 10, 25, 50, and 100 nM DHT. The cells were collected for Western blot analysis **(A)** to determine the IGF1R **(B, C)** and FSHR **(D, E)** protein levels after 12 and 24 h. n = 3 in each time point and each group; one-way ANOVA. *P < 0.05, **P < 0.01.

As shown in **Figures 6A–C**, IGF1R expression of KGN cells increased with androgen concentration in the range of 0–10 nM, while IGF1R expression decreased with androgen concentration in the range of 10–100 nM. The trend of FSHR expression was similar to that of IGF1R after 12 and 24 h of treatment (**Figures 6D, E** **)**. IGF1R and FSHR both reached the highest expression with 10-nM DHT treatment and were significantly higher than those in the control groups after 24 h of treatment. The expression of IGF1R and FSHR was lowest in the 100-nM groups, which was significantly lower than that in the 10-nM groups.

### Androgens enhanced the effect of insulin-like growth factor 1 and follicle-stimulating hormone in promoting the expression of steroid hormone synthase in the presence of high-dose glucocorticoids

**Figure 7:**
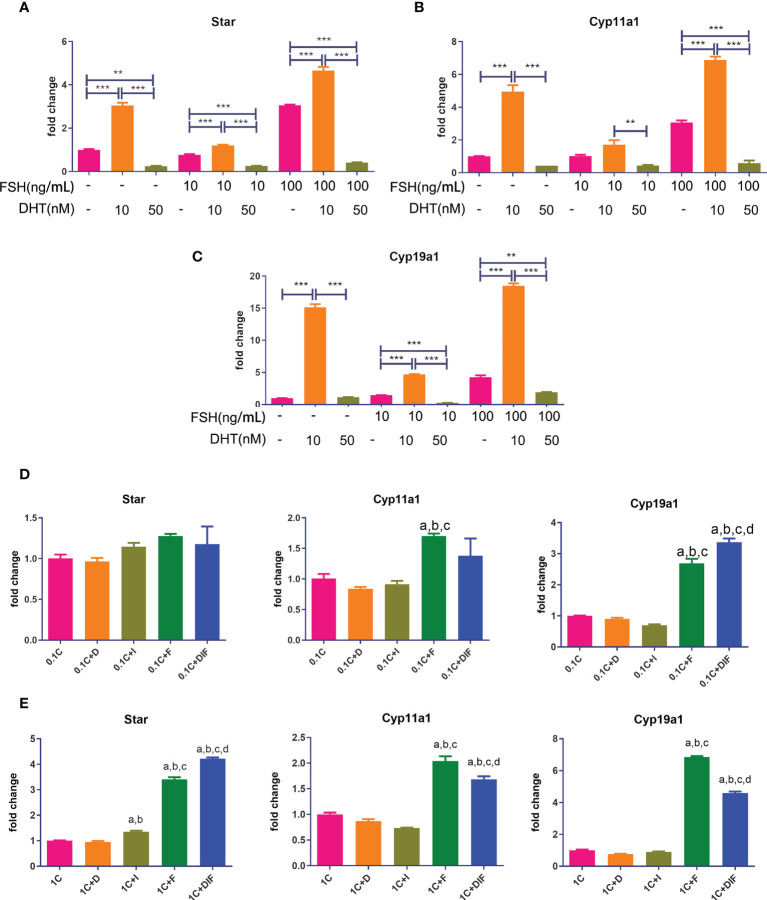
Androgen-IGF1-FSH synergistic effect on promoting the expression of steroid hormone synthase and the influence of glucocorticoids on this effect. KGN cells were treated with solvent or low-dose (10 nM) and high-dose (50 nM) DHT in the presence of different doses of FSH for 24 h mRNA expressions of *Star* **(A)**, *Cyp11a1* **(B)**, and *Cyp19a1* **(C)** were determined by qPCR. n = 3; one-way ANOVA. KGN cells were treated with low-dose **(D)** and high-dose **(E)** CORT for 24 h and then treated with and without DHT, IGF1, or FSH alone and combined DHT-IGF1-FSH, respectively, for 48 h mRNA expressions of *Star*, *Cyp11a1*, and *Cyp19a1* were determined by qPCR. n = 3; one-way ANOVA. 1C: 1 µM CORT; 1C+D: 1 µM CORT + 10 nM DHT; 1C+I: 1 µM CORT + 100 ng/ml IGF1; 1C+F: 1 µM CORT + 20 ng/ml FSH; a: P < 0.05 compared to “1C” group; b: P < 0.05 compared to “1C+D” group; c: P < 0.05 compared to “1C+I” group; d: P < 0.05 compared to “1C+F” group. **P<0.01;***P<0.001.

As shown in **Figures 7A–C**, all of the gene expressions in cells treated with low levels of DHT and FSH at various doses were significantly higher compared to those in cells treated with FSH alone, while gene expressions in cells treated with high levels of DHT and FSH were significantly lower than those in cells treated with FSH alone.

**Figures 7D, E** show the expressions of steroid hormone synthase after treatment with low/high concentrations of CORT followed by either one of the three factors or the combination of the three factors. The expression of Cyp19a1 and Star in cells treated with the combination of DHT-IGF1-FSH was significantly higher than that of cells treated without the three factors or cells, treated with one factor regardless of the dose of CORT. For the expression of other steroid hormone synthases, the results in the combined treatment groups were also significantly higher than those in the cells treated without factors or treated with androgens or IGF1 alone.

### Androgens improved insulin-like growth factor 1 receptor and follicle-stimulating hormone receptor expression of granulosa cells by directly binding to the promoters of *Igr1r* and *Fshr*

**Figure 8:**
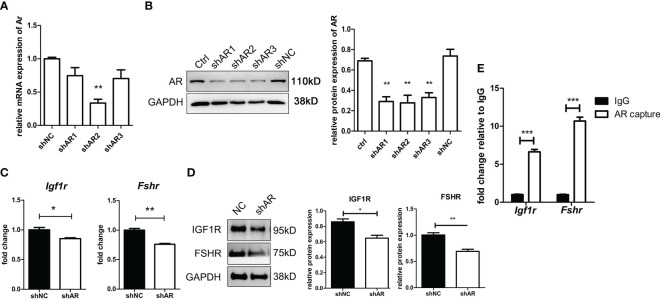
AR directly stimulates IGF1R and FSHR expression by binding to the promoter regions of IGF1R and FSHR. KGN cells were transfected by three shAR vectors. The efficiency of knockdown was tested by qPCR **(A)** and Western blot **(B)**. n = 3; t-test comparison between the control group and other groups. shAR2 was used for further experiments, and the transfected cells were collected to determine the mRNA expression **(C)** and protein expression **(D)** of IGF1R and FSHR. n = 3; t-test. KGN cells were treated with DHT for 1 h followed by the ChIP assay. AR antibody-enriched DNA fragments were detected for promoter sequences of IGF1R and FSHR by qPCR. n = 3; t-test. **(E)** *P < 0.05; **P < 0.01; ***P < 0.001.

As shown in **Figures 8A, B**, the efficiency of three siRNAs was tested using qPCR and WB. The expression of AR in the shAR2 group showed good efficiency in both qPCR and WB results. Therefore, shAR2 was applied in subsequent experiments. The gene and protein expressions of IGF1R and FSHR were significantly downregulated in AR-knockdown KGN cells compared to those of the control group (**Figures 8C, D** **)**. ChIP-qPCR results (**Figure 8E**) showed that the relative enrichment degree of two sequences was significantly higher than those in the IgG negative control, thus confirming the combination of ARs to the promoter regions of *Igf1r* and *Fshr*.

### High-dose glucocorticoids inhibited the growth of mouse ovarian follicles cultured *in vitro*, which was improved by androgens at appropriate doses

**Figure 9:**
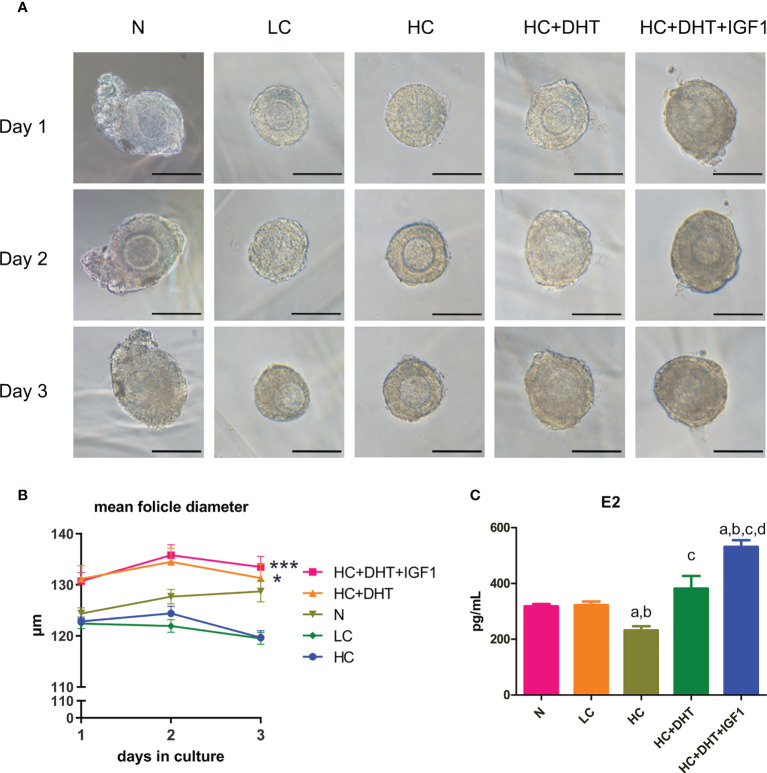
The effect of glucocorticoids on the development of 3D cultured mouse ovarian follicles and the therapeutic effect of androgens. Mouse ovarian follicles were isolated and 3D-cultured in alginate beads for 3 days. The follicles were divided into normal (N), 0.1 µM CORTN (LC), 1 µM CORTN (HC), HC + 10 nM DHT (HC+DHT), and HC + 10 nM DHT + 100 ng/ml IGF1 (HC+DHT+IGF1) groups. The culture medium was supplied with and without listed drugs, respectively. The morphology **(A)** and average diameter **(B)** of follicles in each group were monitored for 3 days. n ≥ 30 in each group; repeated-measures ANOVA. After 3 days of culture, culture medium supernatant was collected for ELISA assay to determine the E2 concentration. n ≥ 7 in each group; one-way ANOVA. **(C)** *P < 0.05 compared to group “N”; ***P < 0.001 compared to group “N”; a: P < 0.05 compared to group “N”; b: P < 0.05 compared to group “LC”; c: P < 0.05 compared to group “HC”; d: P < 0.05 compared to group “HC+DHT.”.

As shown in **Figures 9A, B**, the follicles supplemented with low-dose CORTN (LC) or high-dose CORTN (HC) grew slower than those in the control groups (N). Follicles treated with both 10-nM DHT and high-dose CORTN (HC+DHT) grew significantly faster than those in the HC group. DHT combined with IGF1 treatment (HC+DHT+IGF1) also significantly improved follicle growth, and the growth trend of the follicles was superior to that of the HC+DHT group. E2 concentration was detected by ELISA after a 3-day *in vitro* culture. As shown in **Figure 9C**, E2 concentration in the LC group was not significantly changed compared to that of the N group, but the E2 concentration in the HC group was significantly decreased over that in the N group. E2 concentration in the HC+DHT group was significantly improved compared to that of the HC group, and E2 concentration in the HC+DHT+IGF1 group was significantly higher than those of any other group.

## Discussion

DOR is an important pathological factor affecting female reproductive health and pregnancy rate. An important contributor to DOR, chronic stress adversely affects ovarian reserve mainly through high concentrations of glucocorticoids released after HPA axis activation. In this study, we first investigated the effects of glucocorticoids on apoptosis, cell viability, and E2 secretion in human granulosa cell lines, KGN cells, and mouse primary granulosa cells. We found that high concentrations of glucocorticoids reduced granulosa cell viability and steroid hormone secretion, and the adverse effects increased with glucocorticoid concentrations and duration of intervention. Similarly, in a previous *in vivo* mouse study, Gao et al. (37) found that the oocyte developmental potential of growing follicles decreased with the increase of chronic stress time and intensity. These results suggest that attention be paid to the impairment of long-term chronic stress on ovarian function. When interpreting clinical studies on stress that have conflicting results, special attention should be given to distinguishing between different effects caused by acute and chronic stress, which may lead to different trends in patient serum E2 levels (38, 39). In the results of cell apoptosis, we found that glucocorticoids did not directly induce apoptosis in granulosa cells, which was different from a previous study in mice that found that chronic stress promotes of cumulus cells and granulosa cells in mice (40). This difference in results suggests that chronic stress states may not directly damage granulosa cells but indirectly damage follicle development through certain changes in the ovarian microenvironment. Notably, we observed a significant decrease in T secretion following chronic administration of high-dose glucocorticoids in mouse TIC. To our knowledge, this is the first evidence that chronic stress may affect steroid hormone synthesis of theca cells. Because the binding signal of androgens to ARs is an important factor supporting early follicular development, high levels of glucocorticoids caused by chronic stress can significantly reduce the supporting effect of androgens on early follicular development; concurrently, the precursors of E2 are also reduced, which indirectly leads to ovarian follicle dysfunction. However, the specific mechanism through which glucocorticoids reduce androgen production requires further study.

The expression level of FSHR in granulosa cells determines the sensitivity of follicles to FSH signaling. Although early follicles are gonadotropin-independent, certain concentrations of FSH are required (35). In a mechanistic exploration, we examined the inhibition of FSHR protein expression in KGN cells by prolonged exposure to high-dose glucocorticoids. This result is consistent with our previous reports in a mouse model of unpredictable chronic stress-induced DOR (14, 15). We exposed mice to randomly imposed stressors such as restraint, day-night reversal, single-cage feeding, and tail suspension daily for 8 weeks. We found that the follicular reserve of mice was significantly reduced, so was the protein expression of FSHR in ovarian follicles. In a recent study of primary cultured rat granulosa cells, Kashino et al. (41) found that dexamethasone treatment dose-dependently decreased E2 production induced by FSH and cAMP synthesis induced by FSH. This decreased FSHR signaling may suggest a decreased FSHR expression. Another study in weaned sows found that long-term repeated intravenous administration of adrenocorticotropin hormone, the hormone promoting glucocorticoid secretion, affects E2 secretion and reduced LHR mRNA expression in the corpus luteum (42). Unfortunately, the FSHR expression in follicles remains unknown.

IGF1 signaling is another important growth signal for follicular development, which can regulate E2 production independently or through synergistic effects with FSH (43). Although IGF1R signaling is important for maintaining follicle development and hormone secretion, there are very few previous reports on the effects of excess glucocorticoids or chronic stress on IGF1 and IGF1R expression. An old study in bovine reported that cortisol at physiological levels had little or no effect on the number of IGF1R in granulosa cells from small follicles (44). In this study, we assessed the secretion of IGF1 after glucocorticoid treatment and found no significant differences between groups. However, we found that the autophosphorylation level of IGF1R protein in KGN cells decreased significantly after prolonged exposure to high concentrations of CORT, while the expression of IGF1R did not change significantly. We did not observe identical changes as in KGN cells after treatment of mGC with CORTN. The results showed directly decreased IGF1R protein expression after high concentrations of CORTN intervention. Although there existed this difference in human and mouse cells, the results suggest that chronic stress-induced high concentrations of glucocorticoids impair granulosa cell sensitivity to IGF1 signaling.

Because IGF1 mainly regulates hormone secretion by binding to IGF1R to activate downstream AKT and ERK signaling (10, 45, 46), we also tested whether glucocorticoids themselves can affect the phosphorylation levels of AKT and ERK signaling in KGN cells; results found no significant difference, indicating that high concentrations of glucocorticoids reduced IGF1 signaling sensitivity and E2 secretion by inhibiting IGF1R autophosphorylation in KGN cells. Autophosphorylation of IGF1R occurs on the cell membrane (47); therefore, we hypothesized that when additional ligands bind to GRs under chronic stress, GRs may interact with IGF1R on the cell membrane and thus affect the exposure of IGF1R phosphorylation sites. The co-IP results demonstrate for the first time that GRs and IGF1R interact in KGN cells. This interaction may sustain a certain level of autophosphorylation of IGF1R. Long-time manipulation of high-dose glucocorticoids could weaken the interaction of the two receptors and thus interfere with the autophosphorylation of IGF1R in KGN cells, which leads to the low responsiveness to IGF1 signals. However, the specific protein conformational and phosphorylation site changes during this process remain to be verified.

Treatment for DOR includes various ovarian stimulation regimens and adjuvant sequential hormone therapy. Androgen supplementation is the first treatment option that promises to increase the pool of recruitable follicles (48). Several clinical findings suggest that supplementation with T or DHEA significantly improves ovarian reserve and increases oocyte retrieval and pregnancy rates in DOR patients (24). However, some clinical studies have concluded that androgen supplementation is ineffective (49), which may be attributable to differences in androgen type, dose, and duration of treatment used in different studies. T and DHT are the only two androgens that bind directly to ARs (50). We used DHT in our cellular experiments for this study to exclude T from converting to estrogen and acting through estrogen receptors. We first observed the effect of different doses of DHT on the expression of IGF1R and FSHR on KGN cells. The results showed that androgens had opposite effects at different doses, and only a relatively low dose range (10–25 nM) of androgens could promote the protein expression of IGF1R in KGN cells. This result reemphasizes that maintaining the balance of androgen concentrations is critical for ovarian follicle development (17). A recent study on AR metabolism in granulosa cells showed that ligand binding of ARs significantly prolongs AR half-life by maintaining its nuclear localization and protecting it from degradation in the cytoplasm (51). This positive feedback effect may ensure androgen function at low secretion levels.

We treated KGN cells with DHT and FSH to observe the regulatory effect of different concentrations of androgens on FSH signaling. By examining the mRNA expression of steroid hormone synthase in KGN cells, we found that the effect of androgens on FSH signaling is also bidirectional. Only a low dose range of DHT enhanced the effect of FSH in promoting steroid hormone synthase transcription. Furthermore, both in the context of low- and high-concentration glucocorticoids, androgens in the low-dose range synergized with IGF1 and FSH to promote steroid hormone synthase transcription. These results suggest that appropriate levels of androgens are expected to enhance E2 secretion in granulosa cells under chronic stress conditions through synergistic signaling with IGF1 and FSH.

In the ChIP-qPCR results, we verified that ARs act as transcription factors that directly bind to the *Igf1r* and *Fshr* promoters in KGN cells, thereby regulating the transactivation of the two receptor genes. A recent ChIP sequencing (ChIP-seq) study found that ARs can regulate gene expression by altering the methylation of histones near the gene promoters or enhancers (52). Whether ARs regulate the transcription of *Igf1r* and *Fshr* genes directly or indirectly through the methylation regulation in KGN cells requires future study. However, in mGC, Sen et al. (53) report a different regulatory mechanism of DHT. They demonstrate that 25 nM of DHT promotes FSHR protein expression, but not *Fshr* transcription. They further validated that DHT regulates FSHR through paxillin-activated ERK1/2 signaling, thereby regulating FSHR protein synthesis. This reflects another difference between humans and mice in the mechanism by which androgens regulate the sensitivity of granulosa cells to FSH signaling.

For a more intuitive observation, mouse secondary follicles were isolated and cultured in alginate beads *in vitro*. We observed the effects of glucocorticoids on early follicular development and steroidogenesis and the efficacy of androgens alone and androgens in combination with IGF1. The results confirmed that high concentrations of glucocorticoids inhibited early-stage follicle growth and E2 secretion of ovarian follicles. Androgens at an appropriate dose range can synergize with IGF1 to ameliorate the damage caused by glucocorticoids. To our knowledge, this is the first direct evidence that chronic stress impairs early-stage follicle growth and function in mice. Previous studies have reviewed the effect of different doses of different types of androgens on ovarian follicles cultured *in vitro (* 36, 54). They demonstrate that different types of androgens work best at improving mouse early-stage ovarian follicle development at nearly the same 10-nM concentration. This dose is consistent with the optimal dose of androgens we observed in KGN cell experiments. Therefore, we used this dose of androgens in follicle culture to verify their therapeutic effects. Furthermore, we observed that when IGF1 was added to the culture system, there was a better effect not only on follicle diameter changes but also on E2 secretion. It is worth mentioning that in our pilot study, it was observed that follicle growth was not ideal if FSH was not added to the medium, so the follicle media we used in this study all contained FSH. Based on this fact, we only examined differences between androgen therapy and androgen-IGF1 combination therapy. Our results further validated the synergistic effect of androgens and IGF1.

In conclusion, the present study demonstrates that high-dose glucocorticoids impair E2 secretion in granulosa cells and early-stage ovarian follicles by inhibiting FSHR and IGF1R expression or indirectly by inhibiting autophosphorylation of IGF1R. Appropriate concentrations of androgens can alleviate the damage of high-dose glucocorticoids on follicular growth and steroidogenesis. Androgens can increase ovarian follicle E2 secretion by activating the transcription of IGF1R and FSHR.

## Data availability statement

The original contributions presented in the study are included in the article/supplementary material. Further inquiries can be directed to the corresponding author.

## Ethics statement

This study was reviewed and approved by the Animal Experimental Ethical Committee of Fudan University.

## Author contributions

WW and LG contributed to the conception and design of the study. LG and HG performed the experiments. LG performed the statistical analysis and drafted the manuscript. WW assisted in manuscript modification. All authors contributed to manuscript revision, read, and approved the submitted version.

## Funding

This work was supported by the National Natural Science Foundation of China. (Grant Number: 82074198).

## Conflict of interest

The authors declare that the research was conducted in the absence of any commercial or financial relationships that could be construed as a potential conflict of interest.

## Publisher’s note

All claims expressed in this article are solely those of the authors and do not necessarily represent those of their affiliated organizations, or those of the publisher, the editors and the reviewers. Any product that may be evaluated in this article, or claim that may be made by its manufacturer, is not guaranteed or endorsed by the publisher.
